# The impact of low‐dose aspirin on hemoglobin levels in pregnancy: A secondary analysis of a randomized controlled trial for prevention of hypertensive disorders of pregnancy

**DOI:** 10.1002/ijgo.70710

**Published:** 2025-12-01

**Authors:** N. M. Ngcobo, V. Dorsamy, C. Bagwandeen, P. Z. Mkhize, J. Moodley

**Affiliations:** ^1^ School of Laboratory Medicine and Medical Sciences, College of Health Sciences University of KwaZulu‐Natal Durban South Africa; ^2^ Department of Public Health Medicine, School of Nursing and Public Health University of KwaZulu‐Natal Durban South Africa; ^3^ Women's Health and HIV Research Group, Department of Obstetrics and Gynaecology University of KwaZulu‐Natal Durban South Africa

**Keywords:** anemia, hemoglobin, hypertensive disorders of pregnancy, low‐dose aspirin, preeclampsia

## Abstract

**Objectives:**

This study evaluates the association of low‐dose aspirin (LDA) with hemoglobin (Hb) levels during pregnancy and explores how changes in Hb levels relate to hypertensive disorders of pregnancy (HDP).

**Methods:**

This secondary analysis of a randomized controlled trial comprised 249 pregnant women recruited from a regional hospital in Durban, South Africa (SA). Participants were randomized to receive either 162 mg of LDA daily (intervention) or standard care (control). Hemoglobin levels were measured at enrolment and again at birth. Data on maternal outcomes, including HDP, were analyzed in relation to changes in Hb levels to assess potential associations.

**Results:**

At enrolment, the predominant Hb range was 9.0–10.9 g/dL (mild anemia) in 51.4% of participants, with comparable Hb distributions between groups. By birth, 43.4% remained mildly anemic, while 44.2% had normal Hb levels (11–13 g/dL). Among participants who experienced a decline in Hb, the reduction was significantly mitigated in the intervention compared to the control group (ΔHb = −0.902 vs. −1.422, *P* = 0.002, η^2^ = 0.088). Declining Hb levels increase the odds of developing HDP compared to increasing Hb (odds ratio = 2.29 vs. 0.44, *P* = 0.032). However, the intervention group within this subgroup had 76% reduction in risk of developing HDP (RR = 0.24, 95% confidence interval [0.11–0.53], *P* < 0.001) compared to the control group.

**Conclusion:**

Low‐dose aspirin was associated with a smaller decline in Hb levels and lower HDP risk; consistent with a potential hematological benefit beyond preeclampsia prevention, although this represents an association rather than causation. Hemoglobin reduction might also serve as an early predictor of HDP.

## 1 INTRODUCTION

Low‐dose aspirin (LDA), initiated between 12 and 28 weeks of gestation, is recommended to prevent hypertensive disorders of pregnancy (HDP), which includes gestational hypertension, preeclampsia (PE), and eclampsia, major contributors to adverse maternal and fetal outcomes.^1^ While LDA's role in reducing the risk of HDP is well supported, its potential effects on maternal hemoglobin (Hb) levels remains unclear. Anemia during pregnancy, defined as Hb levels below 11 g/dL in the first or third trimester, is a significant global health issue, especially in low to middle‐income countries (LMICs), where iron deficiency is the most common underlying cause.^2^

Other contributing factors include inherited hematologic conditions such as thalassemia, sickle cell trait, and glucose‐6‐phosphate dehydrogenase (G6PD) deficiency, which are prevalent in parts of sub‐Saharan Africa and can lead to mild to moderate reductions in hemoglobin, increased hemolysis, and potentially altered response to iron supplementation.^3^, ^4^, ^5^ In South Africa (SA), an estimated 31% of pregnant women are affected by anemia, largely due to poor nutrition, infections, micronutrient deficiencies, and, in rare cases, these genetic traits.^6^

There is growing recognition of a bidirectional relationship between anemia and HPD, where HDP‐induced inflammation and oxidative stress might exacerbate anemia, while anemia‐associated hypoxia might impair placental function, increasing the risk of hypertensive complications.^7^, ^8^, ^9^ This interplay highlights the need to assess whether LDA, while reducing HDP risk, might inadvertently compromise maternal Hb levels.

Low‐dose aspirin remains a cost‐effective and widely accessible intervention, particularly valuable in resource‐limited settings. However, its potential iron‐chelating properties and association with gastrointestinal blood loss have raised safety concerns. For instance, Plancha et al.^10^ reported a rare case of severe gastrointestinal bleeding in a pregnant woman taking LDA for HDP prevention. Further, aspirin's metabolites may bind to iron, potentially reducing its bioavailability and impairing iron absorption.^11^, ^12^

Understanding the association of LDA with Hb levels is essential, especially in SSA where both PE and maternal anemia are highly prevalent.^13^, ^14^, ^15^ A prior randomized controlled trial (RCT) in a South African cohort of Black women reported a 75% reduction in HDP risk following LDA administration between 12 and 20 weeks gestation.^16^ However, the trial did not explore hematological outcomes. This secondary analysis aims to address this gap by evaluating whether LDA is associated with changes in Hb levels during pregnancy and how these changes correlate with HDP development. Clarifying this relationship could inform safer clinical practices and maternal health strategies in high‐risk populations.

## 2 MATERIALS AND METHODS

### 2.1 Study design and setting

This study is a secondary retrospective analysis based on maternity case records of participants enrolled in an RCT conducted at a regional hospital in Durban, South Africa. The trial evaluated the efficacy of LDA in preventing HDP and adhered to CONSORT guidelines.^16^, ^17^ It was conducted from 5 May 2021 to 4 March 2024. Pregnant women were randomly assigned to receive either LDA (162 mg daily) until 36 weeks of gestation (intervention) or standard care alone (control). Both groups received routine antenatal care, including regular check‐ups, iron and folic acid supplementation, and multivitamins.

### 2.2 Study population

Normotensive Black pregnant women aged 18 years or older with singleton pregnancies between 12 and 20 weeks of gestation were recruited into the trial. Further, confirmation of a fetal heartbeat and gestational age (GA) within the specified range was required. Exclusion criteria included multiple pregnancies, known fetal abnormalities, pre‐existing hypertension, chronic diseases (e.g., diabetes), prior aspirin use, or contraindications to aspirin.

### 2.3 Randomization and sample size

Participants in the RCT were assigned to either the intervention or control group using a 1:1 allocation ratio. Allocation was determined by coin toss with alternate assignment, ensuring relatively equal group sizes.^16^ While this approach provided group balance, it might have not completely eliminated allocation bias.

### 2.4 Data collection

Hemoglobin levels were measured at enrolment (12–20 weeks) and at prior to birth (on admission). Baseline demographic information, anthropometric measurements, and Hb data were collected using standard hospital laboratory methods with automated hematology analyzers (full blood count) as part of the RCT. Birth Hb levels were retrieved from maternity records or the National Health Laboratory Service (NHLS) if missing. Hypertensive disorders of pregnancy were defined per International Society for the Study of Hypertension in Pregnancy (ISSHP) criteria: gestational hypertension (new‐onset hypertension after 20 weeks of pregnancy with organ involvement), PE (hypertension with proteinuria or organ dysfunction), and PE with severe features such as headache, nausea, vomiting, or eclampsia.^18^

### 2.5 Missing data

Of the 423 participants enrolled in the RCT, data were analyzed for 249 participants. The remaining 174 were excluded due to missing Hb data at birth. As missing cases were evenly distributed between groups, a complete case analysis was performed to minimize potential bias.

### 2.6 Data analysis

Data were analyzed using Statistical Package for the Social Sciences (SPSS) version 29 (IBM, Armonk, NY, USA). Descriptive statistics summarized baseline characteristics. Hemoglobin levels were classified per World Health Organization criteria: normal (11–13 g/dL), mild anemia (9–10.9 g/dL), moderate anemia (7–8.9 g/dL), and high (>13 g/dL).^2^ Repeated‐measures analysis of variance (ANOVA) assessed mean hemoglobin change (ΔHb) between enrolment and birth. Subgroup analyses compared participants with declining versus increasing Hb. Logistic regression examined the association between ΔHb and HDP. Risk of HDP was compared between the intervention and control groups. A *P*‐value < 0.05 was considered statistically significant.

### 2.7 Ethical considerations

Ethical approval was obtained from the University of KwaZulu‐Natal (UKZN) Biomedical Research Ethics Committee (BREC reference BREC/00007156/2024). Informed consent was obtained from all participants during enrolment. Data was stored securely, and the secondary analysis did not require additional consent.

## 3 RESULTS

A total of 249 participants were analyzed, with 125 (50.2%) in the intervention group and 124 (49.8%) in the control group. Their sociodemographic characteristics and baseline characteristics including gravidity, employment, marital status, human immunodeficiency virus (HIV) status and body mass index (BMI) are summarized in Table 1.

**TABLE 1:** Summary of sociodemographic profile and baseline characteristics.

Variables and *P*‐values	Control (*N* = 124)	Intervention (*N* = 125)	Total (*N* = 249)
Gravidity (*P* = 0.17)			
Primigravid	47 (37.9%)	37 (29.6%)	84 (33.7%)
Multigravid	77 (62.1%)	88 (70.4%)	165 (66.3%)
Employment (*P* = 0.10)			
Unemployed	87 (70.2%)	98 (78.4%)	185 (74.3%)
Employed	16 (12.9%)	17 (13.6%)	33 (13.3%)
Student	21 (16.9%)	10 (8.0%)	31 (12.4%)
Marital status (*P* = 0.45)			
Single	101 (81.5%)	97 (77.6%)	198 (79.5%)
Married/Stable	23 (18.5%)	28 (22.4%)	51 (20.5%)
HIV status (*P* = 0.15)			
Negative	70 (56.5%)	59 (47.2%)	129 (51.8%)
Positive	54 (43.5%)	66 (52.8%)	120 (48.2%)
BMI group (*P* = 0.32)			
<30 (Normal)	78 (62.9%)	69 (55.2%)	147 (59.0%)
≥30 (Obese)	46 (37.1%)	56 (44.8%)	102 (41.0%)

*Note*: *p*‐values indicate between‐group comparisons for each variable.

Abbreviations: BMI, body mass index; HIV, human immunodeficiency virus.

### 3.1 Hemoglobin levels at enrolment and at birth

At enrolment, 51.4% (*n* = 128) of participants had mild anemia, with no notable differences between groups. High Hb (0.4%) and moderate anemia (1.2%) were uncommon at enrolment but increased to 4.4% (*n =* 11) and 8.0% (*n* = 20), respectively at birth. Normal Hb levels (44.2%) were most common at birth, followed by mild anemia (43.4%). Detailed distributions are presented in Table 2.

**TABLE 2:** Hemoglobin categories.

Diagnostic criteria and *P*‐values	Control (*N* = 124)	Intervention (*N* = 125)	Total (*N* = 249)
Enrolment Hb (*P* = 0.12)			
High Hb (>13 g/dL)	1 (0.8%)	0 (0.0%)	1 (0.4%)
Normal Hb	51 (41.1%)	66 (52.8%)	117 (47.0%)
Mild anemia	71 (57.3%)	57 (45.6%)	128 (51.4%)
Moderate anemia	1 (0.8%)	2 (1.6%)	3 (1.2%)
Birth Hb (*P* = 0.26)			
High Hb (>13 g/dL)	4 (3.2%)	7 (5.6%)	11 (4.4%)
Normal Hb	51 (41.1%)	59 (47.2%)	110 (44.2%)
Mild anemia	56 (45.2%)	52 (41.6%)	108 (43.4%)
Moderate anemia	13 (10.5%)	7 (5.6%)	20 (8.0%)

*Note*: *p*‐values indicate between‐group comparisons for each Hb category.

### 3.2 Changes in hemoglobin levels from enrolment to childbirth

Overall, Hb levels showed a small mean increase from enrolment to birth in both groups. Repeated‐measures ANOVA assessing differences in Hb levels over time and between groups indicated a non‐significant main effect of time (*F*(1,247) = 2.578, *P* = 0.110, η^2^ = 0.010), suggesting that the observed increase in Hb was not significant. The time × group interaction was also not significant (*F*(1,247) = 1.671, *P* = 0.197, η^2^ = 0.007), indicating that LDA had no effect on mean Hb change over time (Table 3).

**TABLE 3:** Changes in hemoglobin levels from baseline to follow‐up in the overall population.

Group	Sample size	Baseline Hb, Mean ± SD (g/dL)	Follow‐up Hb, Mean ± SD (g/dL)	ΔHb (g/dL)	*p*‐Value (time × group interaction)
Control	124	10.685 ± 0.8466	10.758 ± 1.3959	0.073	0.197
Intervention	125	10.758 ± 0.7312	11.026 ± 1.2305	0.268	
Total	249	10.722 ± 0.790	10.893 ± 1.320	0.171	

Abbreviations: Hb, hemoglobin; SD, standard deviation; ΔHb, mean change in hemoglobin from baseline to follow‐up.

Table 4 presents a significant main effect of time (*F*(1,109) = 211.132, *P* < 0.001, η^2^ = 0.660), indicating a decrease in Hb levels from enrolment to birth. The time × group interaction was also significant (*F*(1,109) = 10.571, *P* = 0.002, η^2^ = 0.088), suggesting that the intervention group experienced a smaller decline in Hb levels compared to the control group (ΔHb −0.902 vs. −1.422 g/dL).

**TABLE 4:** Change in hemoglobin levels from baseline to follow‐up among participants whose Hb decreased, by study group (*n* = 111).

Group	Sample size	Baseline Hb, Mean ± SD (g/dL)	Follow‐up Hb, Mean ± SD (g/dL)	ΔHb (g/dL)	*p*‐Value (time × group interaction)
Control	55	11.111 ± 0.8359	9.689 ± 1.0367	−1.422	0.002
Intervention	56	11.020 ± 0.5616	10.118 ± 0.9092	−0.902	
Total	111	11.065 ± 0.7091	9.905 ± 0.9936	−1.160	

Abbreviations: Hb, hemoglobin; SD, standard deviation; ΔHb, mean change in hemoglobin from baseline to follow‐up.

Similarly, among participants whose Hb levels increased (Table 5), there was a significant main effect of time (*F*(1,136) = 179.990, *P* < 0.001, η^2^ = 0.570), confirming that Hb levels increased over the course of pregnancy. However, the time × group interaction was not significant (*F*(1,136) = 0.345, *P* = 0.560, η^2^ = 0.003), indicating that the pattern of Hb increase did not differ between the control and intervention groups.

**TABLE 5:** Change in hemoglobin levels from baseline to follow‐up among participants whose Hb increased, by study group (*n =* 138).

Group	Sample size	Baseline Hb, Mean ± SD (g/dL)	Follow‐up Hb, Mean ± SD (g/dL)	ΔHb (g/dL)	*p*‐Value (time × group interaction)
Control	69	10.346 ± 0.6917	11.677 ± 1.4432	1.331	0.56
Intervention	69	10.545 ± 0.7858	11.764 ± 0.9280	1.219	
Total	138	11.446 ± 0.740	11.720 ± 1.210	1.274	

Abbreviations: Hb, hemoglobin; SD, standard deviation; ΔHb, mean change in hemoglobin from baseline to follow‐up.

### 3.3 Association between ΔHb, hypertensive disorders of pregnancy, and potential confounders

Logistic regression analysis was performed to assess the association between ΔHb and HDP, adjusting for potential confounders (gestational age, BMI, and HIV status) as presented in Table 6. Mean Hb change remained a significant predictor of HDP; a decline in Hb levels was associated with higher odds of developing HDP (odds ratio [OR] = 2.29, 95% confidence interval [CI] [1.08–4.89], *P* = 0.032), whereas an increase in Hb levels reduced the odds (OR = 0.44, 95% CI [0.21–0.93], *P* = 0.032). Gestational age was the only significant confounder (OR = 0.68, 95% CI [0.60–0.77], *P* < 0.001), while neither BMI (OR = 1.09, 95% CI [0.51–2.33], *P* = 0.835) nor HIV status (OR = 0.88, 95% CI [0.41–1.85], *P* = 0.730) were statistically significant. Within the declining Hb subgroup, the intervention group had a 76% lower risk of HDP compared with the controls (RR = 0.24, 95% CI [0.11–0.53], *P* < 0.001). Additional regression analyses examining ΔHb in relation to gestational age, BMI, HIV status, and group status showed no significant associations.

**TABLE 6:** Association between mean hemoglobin change and hypertensive disorders of pregnancy (adjusted for confounders).

Predictor	OR/RR	95% CI	*p*‐Values
ΔHb (increase)	0.44	0.21–0.93	0.032
ΔHb (decline)	2.29	1.08–4.89	0.032
Gestational age	0.68	0.60–0.77	<0.001
BMI	1.09	0.52–2.33	0.835
HIV status	0.88	0.41–1.85	0.730
LDA group (ΔH decline subgroup)	0.24	0.11–0.53	<0.001

Abbreviations: BMI, body mass index; CI, confidence interval; LDA, low‐dose aspirin; OR, odds ratio; RR, relative risk; SD, standard deviation; ΔHb, mean change in hemoglobin from baseline to follow‐up.

## 4 DISCUSSION

This secondary analysis evaluated the relationship between LDA and Hb changes during pregnancy, as well as the association between ΔHb and HDP. Overall Hb levels showed small mean increases from enrolment to delivery in both groups, with no significant time × group interaction (control: ΔHb +0.073 g/dL; LDA: ΔHb +0.268 g/dL; *P* = 0.197; Table 3), aligning with findings from a secondary analysis of the ASPIRIN trial.^19^

Stratified analysis revealed that among women with declining Hb levels, the reduction was significantly smaller in the LDA group compared with controls (ΔHb −0.902 vs. −1.422 g/dL; *P* = 0.002; Table 4), while no differences were observed between groups among those with increasing Hb (ΔHb +1.219 vs. +1.331 g/dL; *P* = 0.560; Table 5). This suggests that LDA might attenuate Hb decline in women predisposed to falling Hb, possibly through its anti‐inflammatory downregulation of the interleukin 6‐hepcidin pathway, improving iron mobilization and availability for erythropoiesis.^20^, ^21^, ^22^ Enhanced endothelial function and placental perfusion might also contribute by reducing hemodilution and stabilizing Hb levels.^23^ These mechanisms, however, are hypothesis‐generating and were not directly tested. Measuring Hb on admission before labor minimized potential HDP‐related hemoconcentration.

Logistic regression showed that a decline in Hb levels was associated with higher odds of developing HDP (OR = 2.29, 95% CI [1.08–4.89]), whereas an increase in Hb reduced the odds (OR = 0.44, 95% CI [0.21–0.93]). This aligns with evidence linking anemia to hypertensive complications.^24^, ^25^ For example, Chen et al. demonstrated elevated HDP risk in severely anemic women, while Goodchild et al. found mid‐pregnancy anemia to predict late‐pregnancy prehypertension among South African women.^26^, ^27^ Gestational age at delivery was a significant confounder in an adjusted model, consistent with literature identifying HDP as a major contributor to preterm birth.^28^

Importantly, among women with declining Hb, LDA was associated with a 76% reduction in the risk of HDP (RR = 0.24, 95% CI: 0.11–0.53, *P* < 0.001; Table 6), potentially through its known antiplatelet and anti‐inflammatory effects.^29^, ^30^ While low Hb is linked to adverse outcomes, evidence on the hematological impact of LDA remains limited, as most studies focus on PE and related HDP rather than Hb dynamics.^31^

### 4.1 Limitations

This secondary analysis of a single‐center RCT was not originally powered for hematological outcomes, and 174 of 423 participants were excluded due to missing Hb values at birth, necessitating a complete case analysis. Randomization was by coin toss rather than concealed, computer‐generated allocation, although baseline characteristics were broadly balanced. Adherence to iron and folate supplementation, biochemical markers of iron status, and screening for hemoglobinopathies were not assessed, which might have contributed to Hb variability. Subgroup analyses based on Hb decline or increase were exploratory and might introduce bias. Nonetheless, the analysis benefits from prospectively collected trial data, prespecified measurement timing, and adjusted models accounting for key covariates, providing novel evidence on the hematological associations of LDA in pregnancy.

## 5 CONCLUSION

Our findings suggest that overall Hb changes did not differ significantly between groups; however, women whose Hb declined experienced smaller reductions with LDA and a lower incidence of HDP. These association‐level findings might be particularly relevant in LMICs, where the burdens of anemia and HDP remain high. This study provides early evidence of a potential link between LDA and Hb trajectories, forming a basis for hypothesis generation in future trials.

### 5.1 Future directions

These findings could be strengthened by future larger and multicenter studies incorporating longitudinal monitoring of iron status, supplementation adherence, and hemoglobinopathy screening, and designed to include hematological outcomes pre‐specified alongside HDP endpoints. Investigating possible interactions between LDA and micronutrient therapies might also yield insights for integrated maternal health interventions.

## AUTHOR CONTRIBUTIONS

N.M.N. contributed to the conceptualization and drafting of the article and collaborated with V.D. on data acquisition, analysis, and interpretation. V.D. also provided critical review and revisions. C.B., P.Z.M., and J.M. contributed to reviewing the manuscript and offering critical feedback. All authors read and approved the final manuscript.

## FUNDING INFORMATION

This study was funded by the University of KwaZulu‐Natal (UKZN) College of Health Sciences Scholarship (CHS) and the National Research Foundation Thuthuka Grant (TTK170508230162) in collaboration with the University of KwaZulu‐Natal and Medical Research Council of South Africa (SIR Grant UNS14197).

## CONFLICT OF INTEREST STATEMENT

The authors declare that they have no conflict of interests.

## Data Availability

Research data are not shared.
